# Analysis of the relationship between body mass index and kidney function decline in a middle-aged Japanese population: A population-based retrospective cohort study

**DOI:** 10.1371/journal.pone.0349621

**Published:** 2026-05-21

**Authors:** Miki Nakamura, Chiho Yamazaki, Keiju Hiromura, Atsushi Goto, Kei Hamazaki

**Affiliations:** 1 Department of Public Health, Gunma University Graduate School of Medicine, Maebashi, Japan; 2 Department of Nephrology and Rheumatology, Gunma University Graduate School of Medicine, Maebashi, Japan; 3 Department of Public Health, Yokohama City University School of Medicine, Yokohama, Japan; International University of Health and Welfare, School of Medicine, JAPAN

## Abstract

Japan has one of the highest dialysis prevalence rates worldwide, resulting in substantial economic and health burdens. Although obesity is a known risk factor for the decline of kidney function, the impact of being underweight remains unclear. This study aimed to investigate the relationship between body mass index (BMI), including underweight status, and kidney function decline using health check-up data. We conducted a retrospective cohort study involving Japanese individuals aged 40–74 years in Gunma Prefecture who underwent health checkups in fiscal years 2018 and 2020. The exposure was baseline BMI, which was classified into seven categories. The primary outcome was kidney function decline, defined as a ≥ 30% reduction in estimated glomerular filtration rate (eGFR) over a two-year period. Associations between BMI and kidney function decline were assessed using multivariable logistic regression analysis, adjusting for age, sex, smoking status, alcohol consumption, blood pressure, hemoglobin A1c, lipid profile, and baseline eGFR. A total of 64,970 individuals were included in the final analysis. A U-shaped association was observed between BMI and decline in kidney function. Compared to the reference group, those with obesity (BMI = 30.0–39.9 kg/m²; odds ratio [OR]: 2.05; 95% confidence interval [CI]: 1.31–3.19) and those underweight (BMI = 14.0–18.9 kg/m²; OR: 2.09; 95% CI: 1.43–3.04) had significantly increased risk. The U-shaped association between BMI and kidney function decline suggests that both obesity and underweight status are risk factors. Health guidance should target individuals in both categories to prevent chronic kidney disease.

## Introduction

The number of patients undergoing chronic dialysis in Japan continues to rise annually, reaching 349,700 by the end of 2021, with a population prevalence rate of 2,786.4 per million [1]. According to the United States Renal Data System (USRDS) 2021 report, Japan had the second-highest prevalence of patients undergoing dialysis globally [2]. Approximately 15% of the adult Japanese population (corresponding to 14.8 million individuals) is estimated to have chronic kidney disease (CKD) [3]. The increasing number of patients undergoing dialysis poses serious economic challenges and has become an urgent public health concern. In Japan, dialysis is covered by health insurance, with the government spending approximately $11.6 billion annually [4]. This has imposed a significant strain on the country’s economy. Dialysis also places substantial physical and psychological burdens on patients. The Japanese government’s national health promotion plan, “Health Japan 21,” identifies the reduction of new dialysis cases as a key objective [5]. Early intervention to prevent the onset and progression of CKD is one of the most cost-effective strategies for addressing the aforementioned issue.

To reduce the number of patients requiring dialysis, screening individuals exhibiting early signs of kidney function decline, and ensuring timely access to appropriate treatment and lifestyle interventions are essential. Proteinuria is a well-established risk factor for end-stage kidney disease (ESKD), ^6^ and being easily measurable, serves as a common screening tool and clinical research endpoint [6,7]. However, the number of dialysis patients with nephrosclerosis as the primary disease—often without significant proteinuria—has been increasing [1]. In addition, some patients with diabetic kidney disease experience a decline in kidney function without proteinuria [8,9]. Therefore, relying solely on proteinuria is insufficient for diagnosing kidney impairment. In this study, we focused on the estimated glomerular filtration rate (eGFR), now commonly included in routine health checkups, as a means of identifying early decline in kidney function.

Previous studies conducted outside of Japan have identified hypertension, hyperglycemia, proteinuria, smoking, and obesity as major risk factors for decreased kidney function [10–13]. Analyses of health check-up data in Japan have reported similar risk factors, including hypertension, hyperglycemia, and proteinuria [14]. Numerous studies have examined the association between body mass index (BMI) and declining kidney function. A meta-analysis involving over 5.4 million individuals from the general population reported a linear increase in the risk of eGFR decline with a BMI greater than 25 kg/m² [15]. However, studies focused on the potential impact of being underweight on kidney function are limited, especially in the general middle-aged population. In Japan, the high prevalence of nonobese diabetes and the relatively large proportion of underweight individuals highlight the need to evaluate the association between low BMI and kidney function decline [16]. While these trends are observed nationwide, region-specific approaches are also needed to address local challenges effectively. For instance, the number of new patients starting dialysis in Gunma Prefecture has consistently remained high compared to other regions of Japan [1]. Recognizing and addressing such local challenges is essential for reducing the overall burden of dialysis at the national level.

Therefore, the present study aimed to investigate the relationship between BMI and kidney function decline using health check-up data from Gunma Prefecture. The findings are expected to provide valuable insights for developing effective health guidance strategies to help prevent an increase in new dialysis cases.

## Materials and methods

### Study design

This was a retrospective cohort study.

### Study population

We utilized data from health checkup data of the Gunma Prefecture, Japan, which is located in central Honshu, approximately 100 km north of Tokyo, and has a population of approximately 1.9 million.

Three main public Health Insurance systems are operative in Japan, covering nearly the entire population: employee health insurance (EHI), National Health Insurance (NHI), and late elderly health insurance (LEHI). The EHI and NHI cover individuals aged ≤74 years, while LEHI provides insurance for those aged ≥75 years [17]. The EHI is intended for company employees and their dependents, while the NHI covers people who are not eligible for the EHI, including the self-employed, students, retirees, and the unemployed. As of 2020, the NHI has covered approximately 23.0% of the population in Gunma Prefecture [18].

The dataset used in this study was provided by Gunma Prefecture and comprises annual health checkup data for NHI beneficiaries aged ≥40 years, derived from the Specific Health Checkups program, including clinical measurements and self-reported questionnaire data. We initially identified NHI beneficiaries aged 40–74 years in Gunma Prefecture who underwent health checkups in fiscal year 2018. Among these, participants with available eGFR data for both 2018 and 2020, as well as BMI data from 2018, were included in the analysis. Participants aged 73 and 74 years at baseline were excluded because they were not eligible for follow-up health checkups in 2020 due to transition to a different health insurance system at age 75. In addition, individuals with a baseline BMI < 14 or ≥40 kg/m² (based on previous studies) [19,20] and those who self-reported receiving dialysis during the study period were excluded.

### Outcomes

The eGFR was calculated using the Japanese abbreviated equation recommended by the Japanese Society of Nephrology [21], a modified version of the Modification of Diet in Renal Disease study equation [22].

The primary outcome of this study was kidney function decline, defined as a ≥ 30% reduction in eGFR over two years, because such a decline has been shown to be strongly associated with ESKD and is widely accepted as a surrogate endpoint for CKD progression [23].

### Exposures and other variables

The participants’ height and weight were assessed at baseline, and BMI was calculated as weight (kg) divided by height squared (m²).

The covariates included data from health checkups and self-reported questionnaire. Baseline health checkup data included sociodemographic variables (age and sex); basic clinical measures (systolic and diastolic blood pressure); and laboratory test results (eGFR, hemoglobin A1c [HbA1c], fasting or casual blood glucose, triglycerides, high-density lipoprotein [HDL] cholesterol, low-density lipoprotein [LDL] cholesterol, and urine protein). Urine protein was assessed using a dipstick test. Results of “−” and “±” were classified as negative, and “+”, “2+”, and “3+” were classified as positive. Self-reported data were obtained using a standardized questionnaire administered at the time of the health checkup. Participants were asked about their current use of medications for diabetes, hypertension, and dyslipidemia (yes/no), as well as their medical history of cerebrovascular and cardiovascular diseases and lifestyle factors such as smoking status and alcohol consumption. Detailed information on specific drug classes, such as angiotensin-converting enzyme inhibitors and angiotensin receptor blockers, was not available.

### Statistical analysis

BMI was categorized into seven groups based on previous studies involving Japanese populations [19,20]: 14.0–18.9, 19.0–20.9, 21.0–22.9, 23.0–24.9, 25.0–26.9, 27.0–29.9, and 30.0–39.9 kg/m².

Logistic regression was employed to estimate odds ratios (ORs) and 95% confidence intervals (CIs) for ≥30% decline in eGFR over two years, using the BMI category of 23.0–24.9 kg/m² as the reference.

Model 1: adjusted for age and sex

Model 2: Model 1 was further adjusted for smoking status (yes or no) and alcohol consumption (<20 g/day or ≥20 g/day).

Model 3: Model 2 further adjusted for HbA1c (<6.5% or ≥6.5%), systolic blood pressure (<130 or ≥130 mmHg), triglycerides (<150 or ≥150 mg/dL), HDL (<40 or ≥40 mg/dL), and LDL cholesterol (<120 or ≥120 mg/dL).

Model 4: Model 3 further adjusted for baseline eGFR.

These covariates were selected based on established risk factors for kidney function decline, such as hypertension (systolic blood pressure ≥130 mm Hg) and hyperglycemia (HbA1c ≥ 6.5%) [11,24].

P-values for the linear trends were derived using BMI as a continuous variable. A quadratic association trend was assessed using the model that incorporated both a continuous term and a quadratic term for BMI.

Additionally, a sensitivity analysis was conducted using a model adjusted for HbA1c, systolic blood pressure, triglycerides, HDL cholesterol, and LDL cholesterol as continuous variables.

Missing data were imputed using multiple imputations by the chained equations (MICE) in R, with 100 imputations, after which the analysis was performed.

Participants were classified by age, sex, presence of proteinuria, baseline eGFR, and presence of diabetes, using data with imputed missing values, and subgroup analyses were conducted. Diabetes was defined as fasting glucose ≥126 mg/dL, casual glucose ≥200 mg/dL, HbA1c ≥ 6.5%, or current use of anti-diabetic medication.

All statistical analyses were performed using R (version 4.4.1 for Windows).

### Ethics

This study was approved by the Institutional Ethical Review Board of Gunma University (Approval No. HS2023−137) and was conducted in accordance with the principles of the Declaration of Helsinki. The requirement for informed consent was waived because the data were analyzed anonymously. Information about the study was disclosed to the participants through an opt-out procedure. The data were provided by Gunma Prefecture and were used for research purposes between January 2024 and June 2025. The authors did not have access to information that could identify individual participants at any stage of the study.

## Results

### Participants

**Fig 1:**
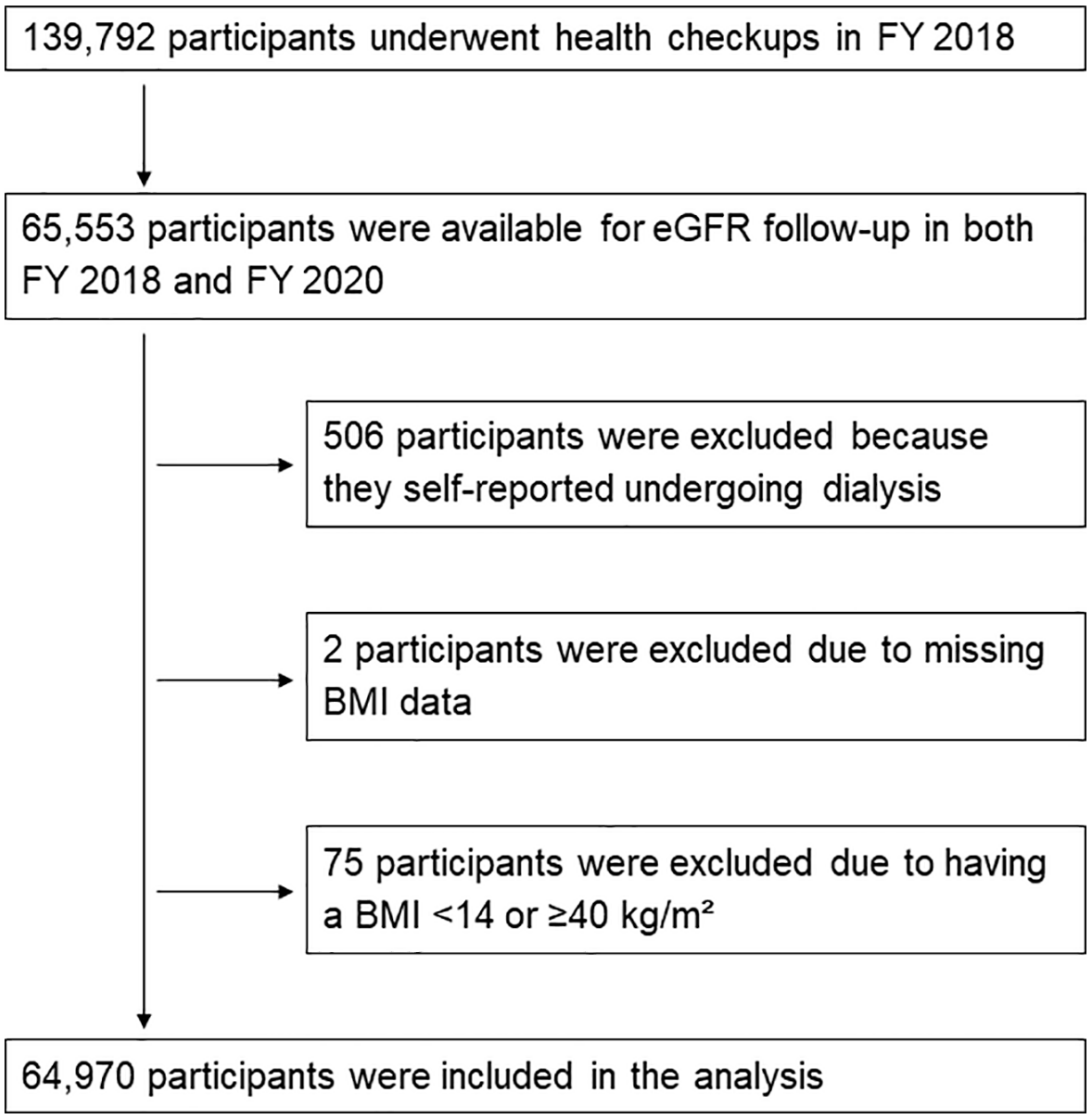
Participants flow. Of the 139,792 National Health Insurance beneficiaries in Gunma screened in fiscal year 2018, 65,553 had follow-up eGFR data for the fiscal years 2018 and 2020. After excluding 506 patients on dialysis, two with missing BMI, and 75 with BMI < 14 or ≥40 kg/m², 64,970 were included in the analysis. eGFR, estimated glomerular filtration rate; BMI, body mass index.

Among the 139,792 NHI beneficiaries in Gunma who underwent health checkups in the fiscal year 2018, 65,553 had follow-up eGFR data available for both fiscal years 2018 and 2020. A total of 506 individuals who self-reported undergoing dialysis were excluded. Additionally, two participants with missing BMI data and 75 with a BMI < 14 or ≥40 kg/m² were excluded. Ultimately, 64,970 individuals were included in the final analysis (Fig 1).

The baseline characteristics of the participants categorized by BMI are presented in Table 1. The mean age was 65.7 ± 6.7 years, with 42.4% being male. The mean BMI was 23.2 ± 3.4 kg/m², with approximately two-thirds of participants falling within the 21.0–26.9 kg/m² range. The higher BMI groups exhibited elevated values of HbA1c, fasting blood glucose, blood pressure, and triglycerides. These groups also had an increased prevalence of medication use for diabetes, hypertension, and dyslipidemia, as well as a significant history of cardiovascular disease.

**Table 1:** Baseline characteristics of participants based on BMI categories.

	BMI category, kg/m^2^							Total
	14.0-18.9	19.0-20.9	21.0-22.9	23.0-24.9	25.0-26.9	27.0-29.9	30.0-39.9	
Number of participants	5615	11003	16370	14922	9168	5639	2253	64970
Age (mean, SD)	65.2 (7.0)	65.5 (6.9)	66.0 (6.5)	66.2 (6.3)	65.9 (6.6)	65.3 (7.1)	63.2 (8.3)	65.7 (6.7)
Sex – Male (n, %)	1146 (20.4)	3406 (31.0)	6943 (42.4)	7440 (49.9)	4879 (53.2)	2792 (49.5)	928 (41.2)	27534 (42.4)
BMI (mean, SD)	17.8 (1.0)	20.1 (0.6)	22.0 (0.6)	23.9 (0.6)	25.8 (0.6)	28.2 (0.8)	32.3 (2.1)	23.2 (3.4)
Smoking (n, %)								
Yes	594 (10.6)	1185 (10.8)	1940 (11.9)	1804 (12.1)	1189 (13.0)	700 (12.4)	251 (11.1)	7663 (11.8)
No	5019 (89.4)	9817 (89.2)	14430 (88.1)	13117 (87.9)	7979 (87.0)	4939 (87.6)	2002 (88.9)	57303 (88.2)
Alcohol consumption (n, %)								
<20 g/day	4945 (88.1)	9250 (84.1)	13026 (79.6)	11584 (77.6)	7032 (76.7)	4501 (79.8)	1914 (85.0)	52252 (80.4)
≥20 g/day	608 (10.8)	1602 (14.6)	3127 (19.1)	3159 (21.2)	2025 (22.1)	1079 (19.1)	318 (14.1)	11918 (18.3)
eGFR, mL/min/1.73m^2^ (mean, SD)	75.6 (14.4)	73.8 (13.9)	72.6 (13.5)	71.8 (14.1)	71.2 (14.8)	71.3 (14.6)	72.9 (15.9)	72.6 (14.2)
HbA1c, % (mean, SD)	5.7 (0.5)	5.7 (0.5)	5.8 (0.6)	5.8 (0.6)	5.9 (0.7)	6.0 (0.8)	6.2 (0.9)	5.8 (0.6)
Fasting blood glucose, mg/dL (mean, SD)	94.4 (16.0)	96.1 (15.5)	98.6 (16.9)	101.1 (18.8)	103.2 (19.6)	105.7 (22.0)	110.4 (27.0)	100.0 (18.7)
Systolic blood pressure, mmHg (mean, SD)	123.7 (17.7)	126.6 (17.0)	129.2 (16.2)	131.4 (15.6)	133.2 (15.6)	134.3 (14.9)	136.1 (15.4)	130.0 (16.4)
Diastolic blood pressure, mmHg (mean, SD)	73.1 (10.8)	74.8 (10.5)	76.2 (10.3)	77.8 (10.2)	78.9 (10.2)	79.5 (10.3)	80.7 (10.5)	76.9 (10.6)
Triglycerides, mg/dL (mean, SD)	83.4 (45.0)	97.6 (55.3)	113.5 (71.6)	127.5 (79.1)	140.4 (86.7)	145.0 (86.1)	147.8 (89.7)	119.1 (75.9)
HDL cholesterol, mg/dL (mean, SD)	76.2 (17.8)	69.5 (16.9)	64.1 (15.7)	59.7 (14.6)	56.9 (13.9)	55.3 (13.3)	54.4 (13.1)	62.9 (16.5)
LDL cholesterol, mg/dL (mean, SD)	119.6 (29.2)	123.0 (29.7)	124.3 (31.1)	124.8 (30.4)	124.2 (30.0)	123.7 (30.6)	122.9 (30.8)	123.7 (30.4)
Urine protein (n, %)								
Negative	5431 (96.7)	10698 (97.3)	15803 (96.5)	14257(95.5)	8623 (94.1)	5208 (92.4)	1988 (88.2)	62008 (95.4)
Positive	179 (3.2)	299 (2.7)	558 (3.4)	659(4.4)	537 (5.9)	428 (7.6)	260 (11.5)	2920 (4.5)
Diabetic medication use (n, %)	243 (4.3)	576 (5.2)	1195 (7.3)	1274 (8.5)	967 (10.5)	810 (14.4)	457 (20.3)	5522 (8.5)
Antihypertensive medication use (n, %)	1000 (17.8)	2697 (24.5)	5343 (32.6)	6132 (41.1)	4627 (50.5)	3230 (57.3)	1458 (64.7)	24487 (37.7)
Lipid medication use (n, %)	756 (13.5)	2190 (19.9)	4145 (25.3)	4287 (28.7)	2993 (32.6)	2062 (36.6)	918 (40.7)	17351 (26.7)
Treatment history (n, %)								
Cerebrovascular disease	109 (1.9)	229 (2.1)	435 (2.7)	452 (3.0)	322 (3.5)	209 (3.7)	74 (3.3)	1830 (2.8)
Cardiovascular disease	201 (3.6)	465 (4.2)	864 (5.3)	865 (5.8)	584 (6.4)	362 (6.4)	168 (7.5)	3509 (5.4)

eGFR, estimated glomerular filtration rate; BMI, body mass index

### BMI and kidney function decline

A ≥ 30% decline in eGFR over two years was observed in 423 participants (0.7%). The OR for this outcome, stratified by BMI and adjusted for covariates, is presented in Table 2. In the fully adjusted model (model 4), a U-shaped association was noted between BMI and the odds of kidney function decline. Participants categorized as obese (BMI = 30.0–39.9 kg/m², OR [95% CI]: 2.05 [1.31–3.19]) and those categorized as underweight (BMI = 14.0–18.9 kg/m², OR [95% CI]: 2.09 [1.43–3.04]) had a significantly higher risk of impaired kidney function compared with the reference group (BMI = 23.0–24.9 kg/m²). Although the linear trend was not statistically significant, the quadratic trend was (P < 0.001), indicating a U-shaped relationship.

**Table 2:** Odds ratios for ≥30% decline in eGFR at 2 years, by BMI categories.

	BMI category, kg/m^2^							P for linear trend	P for quadratic trend
	14.0-18.9	19.0-20.9	21.0-22.9	23.0-24.9	25.0-26.9	27.0-29.9	30.0-39.9		
Number of participants	5615	11003	16370	14922	9168	5639	2253		
Number of events	47	69	92	78	66	43	28		
Proportion, %	0.84	0.63	0.56	0.52	0.72	0.76	1.24		
Odds ratio (95% CI)									
Crude	1.61 (1.12-2.31)	1.20 (0.87-1.66)	1.08 (0.79-1.46)	1.00	1.38 (0.99-1.92)	1.46 (1.01-2.12)	2.39 (1.55-3.70)	0.060	<0.001
Model 1	1.79 (1.24-2.59)	1.29 (0.93-1.79)	1.10 (0.81-1.49)	1.00	1.39 (0.997-1.93)	1.52 (1.04-2.21)	2.77 (1.79-4.28)	0.072	<0.001
Model 2	1.74 (1.21-2.52)	1.27 (0.92-1.76)	1.09 (0.81-1.48)	1.00	1.39 (1.00003-1.93)	1.52 (1.05-2.22)	2.78 (1.80-4.31)	0.049	<0.001
Model 3	2.22 (1.53-3.23)	1.53 (1.10-2.13)	1.20 (0.89-1.63)	1.00	1.27 (0.91-1.77)	1.26 (0.86-1.83)	2.00 (1.28-3.12)	0.403	<0.001
Model 4	2.09 (1.43-3.04)	1.48 (1.07-2.07)	1.19 (0.88-1.62)	1.00	1.28 (0.92-1.78)	1.29 (0.88-1.88)	2.05 (1.31-3.19)	0.664	<0.001

Model 1: adjusted for age and sex.

Model 2: Model 1 further adjusted for smoking (yes or no) and alcohol intake (< 20 g/day or ≥ 20 g/day).

Model 3: Model 2 further adjusted for HbA1c (<6.5% or ≥6.5%), systolic blood pressure (<130 mmHg or ≥130 mmHg), triglycerides (<150 mg/dL or ≥150 mg/dL), HDL cholesterol (<40 mg/dL or ≥40 mg/dL), LDL cholesterol (<120 mg/dL or ≥120 mg/dL).

Model 4: Model 3 further adjusted for baseline eGFR.

eGFR, estimated glomerular filtration rate; BMI, body mass index; HDL, high-density lipoprotein; LDL, low-density lipoprotein; HbA1c, glycated hemoglobin

In the model adjusted with covariates as continuous variables, both obesity and underweight remained significant risk factors for kidney function decline. A significant U-shaped association was observed, and the results were consistent with those of the main analysis (S1 Table). The results were materially unchanged after further adjustment for antihypertensive medication use (S2 Table).

### Subgroup analyses of BMI and kidney dysfunction

The results of the subgroup analyses stratified by age, sex, proteinuria, baseline eGFR, and diabetes are demonstrated in S3 Table. In all subgroups, except those with proteinuria or diabetes, both underweight and obesity were significantly associated with an increased risk of kidney function decline. Furthermore, a significant U-shaped association was observed in all subgroups except for those with proteinuria, as demonstrated by the quadratic trend test.

## Discussion

In this study involving over 60,000 middle-aged Japanese individuals from the general population, we identified a U-shaped association between BMI and kidney function decline. Fully adjusted models revealed that both high and low BMIs were associated with an increased risk of kidney function decline. Subgroup analyses revealed that this pattern persisted even among individuals without pre-existing CKD, such as those without proteinuria or participants with a relatively high baseline eGFR.

Previous studies examining the relationship between BMI and kidney function have generally reported a linear association, and the link between obesity and impaired kidney function has been well established [15,25,26]. Some studies have demonstrated that being underweight serves as a risk factor for impaired kidney function in older individuals [27] and patients with CKD [28–30]. Only a few studies targeting the general population have suggested an association between being underweight and impaired kidney function [31,32]. These studies have certain limitations, including a lack of baseline kidney function data and the use of broad BMI categories. However, this study was able to provide odds ratios adjusted for baseline kidney function and performed analyses with more granular BMI classifications. In Western countries, the proportion of individuals with low BMI is relatively small [33], which may limit the ability to adequately assess the risk associated with being underweight. In a meta-analysis of cohort studies from 40 countries, individuals with a BMI < 18.5 kg/m² were excluded [15]. In contrast, Asian countries, including Japan, exhibit an increased proportion of individuals with low BMI and a lower proportion with high BMI compared to Western countries [33,34]. In the present study, BMI categories were defined to reflect the Japanese population distribution, enabling a more precise evaluation of the risks associated with a low BMI. To the best of our knowledge, this is the first study in the general population to demonstrate that both being underweight and obese are significant risk factors for kidney function decline, with a consistent U-shaped association across all adjusted models.

Obesity is an independent risk factor for proteinuria [6,35]. In cases of obesity-related glomerulopathy diagnosed using renal biopsy, proteinuria is often present and may progress to renal failure [36,37]. Consistent with previous findings, our baseline data demonstrated a much higher prevalence of proteinuria in individuals with a high BMI than in those with a low BMI. However, the rate of eGFR decline increased in both groups. Therefore, assessing the risk of progression to kidney failure based solely on proteinuria may underestimate the risk in individuals with low BMI. Thus, this study provided valuable insights by directly assessing kidney function using eGFR. The definition of kidney function decline used in this study (≥30% decline in eGFR over two years) may capture relatively rapid and clinically meaningful deterioration in kidney function rather than gradual decline. Therefore, the findings may reflect risk factors associated with more rapid kidney function decline in the general population.

Subgroup analyses revealed that underweight individuals aged <65 years and males had a particularly high risk of declining kidney function. Young individuals and males typically possess greater muscle mass, resulting in higher BMI values. Consequently, those with low BMI in these groups may represent individuals with extremely low body weight, potentially explaining the increased risk. Conversely, in participants with diabetes or proteinuria, neither underweight nor obesity was significantly associated with a decline in kidney function. Hyperglycemia plays a major role in the development and progression of diabetic kidney disease [12]. Although obesity is a known risk factor for diabetes, advanced diabetes mellitus can lead to weight loss [38]. In our study, the diabetes status was classified as present or absent, with glycemic control not being evaluated. Therefore, kidney function may have declined due to poor glycemic control regardless of BMI, potentially elucidating the lack of an association. Similarly, the severity of proteinuria influences the kidney prognosis, with heavy proteinuria constituting a significant risk factor [39]. As proteinuria was assessed only as present or absent, we could not account for its severity, potentially obscuring any BMI-related effects on kidney outcomes.

Low body weight is not considered a concern in Japan. According to the World Health Organization, the global mortality burden of noncommunicable diseases, including cardiovascular diseases, diabetes (including diabetic kidney disease), cancer, and chronic lung disease, is rapidly increasing [40]. This highlights the need for a deep understanding of these conditions and the development of effective strategies to address them. As these conditions progress, they can result in weight loss [38,41], making the maintenance and improvement of the health of individuals with low body weight critical. Based on the findings of this study, promoting careful monitoring of kidney function and providing lifestyle guidance for the prevention of CKD, not only among obese individuals, but also among those with low body weight, on a global scale, is essential.

However, the mechanisms underlying the association between underweight status and kidney dysfunction remain unclear. Decreased muscle mass has been reported to be associated with systemic oxidative stress and inflammation [42]. Activation of inflammatory cytokines and chemokines can lead to cell death, thereby contributing to a decline in kidney function [43]. Some individuals with a normal or low BMI may still exhibit metabolic characteristics that are typically associated with obesity [44]. In addition, patients with CKD and low BMI have been reported to have a high incidence of cancer, with previous studies demonstrating an interrelationship between cancer and kidney dysfunction [29]. Although this study lacked data on comorbid conditions, such as cancer [45,46], the possibility that underweight status was related to such complications cannot be excluded.

This study had several limitations. First, the participants were limited to beneficiaries of the NHI system who voluntarily underwent health check-ups. These participants may be more health-conscious than the general population. In addition, participants with follow-up eGFR data were generally healthier than those without follow-up (S4 Table), suggesting potential selection bias. This may have affected the generalizability of the findings. The NHI primarily covers self-employed individuals, retirees, and others not employed by companies. To enhance the generalizability of the findings, future studies should include individuals covered by other public health insurance systems in Japan, such as EHI, as well as individuals of non-Japanese ethnicities. Second, we lacked data on comorbid conditions that may result in low body weight (e.g., cancer) and detailed information on body composition (e.g., muscle mass and fat percentage). Therefore, we cannot completely rule out the possibility that weight loss due to an underlying condition contributed to the decline in kidney function. Third, the follow-up period was relatively short to assess difficult outcomes such as ESKD. Future studies using prolonged follow-up data, including the eGFR slope and incidence of ESKD, are warranted. Finally, as this was an observational study, we could not determine whether weight modification (gain or loss) led to improved kidney outcomes. Future research examining interventions such as dietary modifications and their effects on kidney outcomes would be valuable.

## Conclusions

This study demonstrated a U-shaped relationship between BMI and kidney function decline, indicating that both obesity and being underweight are significant risk factors. To prevent future cases of ESKD, providing health guidance not only to individuals with obesity but also to those who are underweight is important.

## Supporting information

S1 Table Sensitivity analysis of odds ratios for ≥30% decline in eGFR at 2 years, by BMI categories (adjusted for continuous variables). (DOCX)

S2 Table Sensitivity analysis of odds ratios for ≥30% decline in eGFR at 2 years, by BMI categories (further adjusted for antihypertensive medication use). (DOCX)

S3 Table Subgroup analysis. (DOCX)

S4 Table Baseline characteristics of participants with and without follow-up eGFR data in 2020 among individuals with baseline eGFR data in 2018. (DOCX)
