# Supplementary material for: Analysis of the relationship between body mass index and kidney function decline in a middle-aged Japanese population: A population-based retrospective cohort study
Source: PLoS One. 2026 May 21;21(5):e0349621. doi: 10.1371/journal.pone.0349621 (PMC13193553; doi:10.1371/journal.pone.0349621)
Supplement: S1 Table — (DOCX) [file pone.0349621.s001.docx]

**S1 Table. Sensitivity analysis of odds ratios for ≥30% decline in eGFR at 2 years, by BMI categories (adjusted for continuous variables)**

|  | BMI category, kg/m^2^ | | | | | | | P for linear trend | P for quaratic trend |
| --- | --- | --- | --- | --- | --- | --- | --- | --- | --- |
|  | 14.0-18.9 | 19.0-20.9 | 21.0-22.9 | 23.0-24.9 | 25.0-26.9 | 27.0-29.9 | 30.0-39.9 |  |  |
| Number of participants | 5615 | 11003 | 16370 | 14922 | 9168 | 5639 | 2253 |  |  |
| Number of events | 47 | 69 | 92 | 78 | 66 | 43 | 28 |  |  |
| Proportion, % | 0.8 | 0.6 | 0.6 | 0.5 | 0.7 | 0.8 | 1.2 |  |  |
| Odds ratio (95% CI) |  |  |  |  |  |  |  |  |  |
| Crude | 1.61  (1.12-2.31) | 1.20  (0.87-1.66) | 1.08  (0.80-1.46) | 1.00 | 1.38  (0.99-1.92) | 1.46  (1.01-2.12) | 2.39  (1.55-3.70) | 0.060 | <0.001 |
| Model 1 | 1.79  (1.24-2.59) | 1.29  (0.93-1.79) | 1.10  (0.81-1.49) | 1.00 | 1.39  (1.00-1.93) | 1.52  (1.04-2.21) | 2.77  (1.79-4.28) | 0.072 | <0.001 |
| Model 2 | 1.74  (1.20-2.51) | 1.18  (0.85-1.65) | 1.05  (0.77-1.42) | 1.00 | 1.39  (1.00-1.93) | 1.52  (1.05-2.22) | 2.68  (1.72-4.18) | 0.049 | <0.001 |
| Model 3’ | 2.36  (1.60-3.46) | 1.58  (1.13-2.21) | 1.21  (0.89-1.65) | 1.00 | 1.27  (0.91-1.76) | 1.25  (0.86-1.83) | 1.96  (1.26-3.06) | 0.311 | <0.001 |
| Model 4’ | 2.21  (1.51-3.25) | 1.53  (1.10-2.14) | 1.20  (0.88-1.63) | 1.00 | 1.27  (0.91-1.77) | 1.29  (0.89-1.88) | 2.02  (1.30-3.16) | 0.554 | <0.001 |

Model 1: adjusted for age and sex.

Model 2: Model 1 further adjusted for smoking (yes or no) and alcohol intake (< 20 g/day or ≥ 20 g/day).

Model 3’: Model 2 further adjusted for HbA1c, systolic blood pressure, triglycerides, HDL cholesterol and LDL cholesterol as continuous variables.

Model 4’: Model 3’ further adjusted for baseline eGFR.

eGFR, estimated glomerular filtration rate; BMI, body mass index; HDL, high-density lipoprotein; LDL, low-density lipoprotein; HbA1c, glycated hemoglobin
