# Supplementary material for: Analysis of the relationship between body mass index and kidney function decline in a middle-aged Japanese population: A population-based retrospective cohort study
Source: PLoS One. 2026 May 21;21(5):e0349621. doi: 10.1371/journal.pone.0349621 (PMC13193553; doi:10.1371/journal.pone.0349621)
Supplement: S2 Table — (DOCX) [file pone.0349621.s002.docx]

**S2 Table. Sensitivity analysis of odds ratios for ≥30% decline in eGFR at 2 years, by BMI categories (further adjusted for antihypertensive medication use)**

|  | BMI category, kg/m^2^ | | | | | | | P for linear trend | P for quadratic trend |
| --- | --- | --- | --- | --- | --- | --- | --- | --- | --- |
|  | 14.0-18.9 | 19.0-20.9 | 21.0-22.9 | 23.0-24.9 | 25.0-26.9 | 27.0-29.9 | 30.0-39.9 |  |  |
| Number of participants | 5615 | 11003 | 16370 | 14922 | 9168 | 5639 | 2253 |  |  |
| Number of events | 47 | 69 | 92 | 78 | 66 | 43 | 28 |  |  |
| Proportion, % | 0.84 | 0.63 | 0.56 | 0.52 | 0.72 | 0.76 | 1.24 |  |  |
| Odds ratio (95% CI) |  |  |  |  |  |  |  |  |  |
| Model 4' | 2.38  (1.63-3.47) | 1.25  (1.16-2.26) | 1.21  (0.92-1.69) | 1.00 | 1.21  (0.87-1.69) | 1.18  (0.81-1.72) | 1.76  (1.13-2.75) | 0.085 | <0.001 |

Model 4': Model 4 further adjusted for antihypertensive medication use.

eGFR, estimated glomerular filtration rate; BMI, body mass index
