# Supplementary material for: Analysis of the relationship between body mass index and kidney function decline in a middle-aged Japanese population: A population-based retrospective cohort study
Source: PLoS One. 2026 May 21;21(5):e0349621. doi: 10.1371/journal.pone.0349621 (PMC13193553; doi:10.1371/journal.pone.0349621)
Supplement: S3 Table — (DOCX) [file pone.0349621.s003.docx]

**S3 Table. Subgroup analysis**

|  |  | BMI category, kg/m^2^ | | | | | | | P for linear trend | P for quadratic trend |
| --- | --- | --- | --- | --- | --- | --- | --- | --- | --- | --- |
|  |  | 14.0-18.9 | 19.0-20.9 | 21.0-22.9 | 23.0-24.9 | 25.0-26.9 | 27.0-29.9 | 30.0-39.9 |  |  |
| Age | Age <65 |  |  |  |  |  |  |  |  |  |
|  | Number of participants | 1727 | 3206 | 4149 | 3686 | 2416 | 1657 | 937 |  |  |
|  | Number of events | 16 | 15 | 13 | 11 | 8 | 9 | 9 |  |  |
|  | Proportion, % | 0.93 | 0.47 | 0.31 | 0.30 | 0.33 | 0.54 | 0.96 |  |  |
|  | Model 5 | 3.05  (1.36-6.81) | 1.63  (0.73-3.62) | 1.10  (0.49-2.47) | 1.00 | 1.02  (0.41-2.54) | 1.59  (0.65-3.89) | 2.38  (0.95-5.97) | 0.555 | <0.001 |
|  | Age ≥65 |  |  |  |  |  |  |  |  |  |
|  | Number of participants | 3888 | 7797 | 12221 | 11236 | 6752 | 3982 | 1316 |  |  |
|  | Number of events | 31 | 54 | 79 | 67 | 58 | 34 | 19 |  |  |
|  | Proportion, % | 0.80 | 0.69 | 0.65 | 0.60 | 0.86 | 0.85 | 1.44 |  |  |
|  | Model 5 | 1.74  (1.12-2.71) | 1.42  (0.99-2.05) | 1.20  (0.87-1.67) | 1.00 | 1.35  (0.95-1.92) | 1.26  (0.83-1.91) | 1.99  (1.18-3.35) | 0.911 | 0.001 |
| Sex | Male |  |  |  |  |  |  |  |  |  |
|  | Number of participants | 1146 | 3406 | 6943 | 7440 | 4879 | 2792 | 928 |  |  |
|  | Number of events | 13 | 25 | 44 | 44 | 35 | 22 | 16 |  |  |
|  | Proportion, % | 1.13 | 0.73 | 0.63 | 0.59 | 0.72 | 0.79 | 1.72 |  |  |
|  | Model 5 | 2.48  (1.31-4.68) | 1.48  (0.90-2.45) | 1.17  (0.77-1.78) | 1.00 | 1.15  (0.74-1.81) | 1.16  (0.69-1.96) | 2.63  (1.45-4.76) | 0.926 | <0.001 |
|  | Female |  |  |  |  |  |  |  |  |  |
|  | Number of participants | 4469 | 7597 | 9427 | 7482 | 4289 | 2847 | 1325 |  |  |
|  | Number of events | 34 | 44 | 48 | 34 | 31 | 21 | 12 |  |  |
|  | Proportion, % | 0.76 | 0.58 | 0.51 | 0.45 | 0.72 | 0.74 | 0.91 |  |  |
|  | Model 5 | 1.98  (1.21-3.23) | 1.52  (0.97-2.41) | 1.27  (0.82-1.98) | 1.00 | 1.43  (0.87-2.35) | 1.44  (0.83-2.50) | 1.55  (0.79-3.04) | 0.457 | 0.004 |
| Urine protein | Urine protein positive |  |  |  |  |  |  |  |  |  |
|  | Number of participants | 180 | 300 | 559 | 660 | 538 | 428 | 260 |  |  |
|  | Number of events | 5 | 8 | 18 | 21 | 21 | 10 | 16 |  |  |
|  | Proportion, % | 2.78 | 2.67 | 3.22 | 3.18 | 3.90 | 2.34 | 6.15 |  |  |
|  | Model 5 | 1.46  (0.52-4.07) | 1.08  (0.46-2.51) | 1.07  (0.56-2.04) | 1.00 | 1.05  (0.56-1.96) | 0.58  (0.27-1.27) | 1.50  (0.74-3.05) | 0.856 | 0.097 |
|  | Urine protein negative |  |  |  |  |  |  |  |  |  |
|  | Number of participants | 5435 | 10703 | 15811 | 14262 | 8630 | 5211 | 1993 |  |  |
|  | Number of events | 42 | 61 | 74 | 57 | 45 | 33 | 12 |  |  |
|  | Proportion, % | 0.77 | 0.57 | 0.47 | 0.40 | 0.52 | 0.63 | 0.60 |  |  |
|  | Model 5 | 2.13  (1.41-3.23) | 1.61  (1.11-2.33) | 1.26  (0.89-1.79) | 1.00 | 1.24  (0.83-1.84) | 1.50  (0.97-2.31) | 1.38  (0.73-2.59) | 0.106 | <0.001 |
| Baseline eGFR | Baseline eGFR <60 |  |  |  |  |  |  |  |  |  |
|  | Number of participants | 644 | 1556 | 2667 | 2781 | 1896 | 1163 | 417 |  |  |
|  | Number of events | 7 | 15 | 16 | 21 | 24 | 13 | 11 |  |  |
|  | Proportion, % | 1.09 | 0.96 | 0.60 | 0.76 | 1.27 | 1.12 | 2.64 |  |  |
|  | Model 5 | 2.84  (1.14-7.11) | 1.97  (0.98-3.95) | 0.97  (0.49-1.90) | 1.00 | 1.62  (0.88-2.97) | 1.15  (0.56-2.36) | 2.24  (1.02-4.94) | 0.882 | 0.020 |
|  | Baseline eGFR ≥60 |  |  |  |  |  |  |  |  |  |
|  | Number of participants | 4971 | 9447 | 13703 | 12141 | 7272 | 4476 | 1836 |  |  |
|  | Number of events | 40 | 54 | 76 | 57 | 42 | 30 | 17 |  |  |
|  | Proportion, % | 0.80 | 0.57 | 0.55 | 0.47 | 0.58 | 0.67 | 0.93 |  |  |
|  | Model 5 | 1.90  (1.24-2.91) | 1.40  (0.96-2.06) | 1.32  (0.93-1.87) | 1.00 | 1.15  (0.76-1.73) | 1.33  (0.85-2.09) | 1.73  (0.99-3.03) | 0.386 | <0.001 |
| Diabetes | Diabetes |  |  |  |  |  |  |  |  |  |
|  | Number of participants | 384 | 951 | 1928 | 2238 | 1726 | 1416 | 769 |  |  |
|  | Number of events | 8 | 12 | 21 | 30 | 27 | 20 | 17 |  |  |
|  | Proportion, % | 2.08 | 1.26 | 1.09 | 1.34 | 1.56 | 1.41 | 2.21 |  |  |
|  | Model 5 | 2.16  (0.96-4.86) | 1.81  (0.60-2.34) | 0.92  (0.52-1.62) | 1.00 | 1.21  (0.71-2.05) | 1.07  (0.60-1.90) | 1.82  (0.98-3.39) | 0.645 | 0.009 |
|  | Not diabetes |  |  |  |  |  |  |  |  |  |
|  | Number of participants | 5231 | 10052 | 14442 | 12684 | 7442 | 4223 | 1484 |  |  |
|  | Number of events | 39 | 57 | 71 | 48 | 39 | 23 | 11 |  |  |
|  | Proportion, % | 0.75 | 0.57 | 0.49 | 0.38 | 0.52 | 0.54 | 0.74 |  |  |
|  | Model 5 | 2.10  (1.36-3.26) | 1.61  (1.09-2.38) | 1.34  (0.93-1.94) | 1.00 | 1.30  (0.84-2.00) | 1.40  (0.85-2.32) | 1.97  (1.01-3.84) | 0.266 | <0.001 |

Model 5, odds ratio (95% CI): adjusted model excluding the target factors of each subgroup from Model 4.

eGFR, estimated glomerular filtration rate; CI, confidence interval; BMI, body mass index
