# Supplementary material for: Analysis of the relationship between body mass index and kidney function decline in a middle-aged Japanese population: A population-based retrospective cohort study
Source: PLoS One. 2026 May 21;21(5):e0349621. doi: 10.1371/journal.pone.0349621 (PMC13193553; doi:10.1371/journal.pone.0349621)
Supplement: S4 Table — (DOCX) [file pone.0349621.s004.docx]

**S4 Table. Baseline characteristics of participants with and without follow-up eGFR data in 2020 among individuals with baseline eGFR data in 2018**

|  | No follow-up eGFR | Follow-up eGFR available | P-value |
| --- | --- | --- | --- |
| Number of participants | 49347 | 64970 |  |
| Age (mean, SD) | 66.0 (8.8) | 65.7 (6.7) | <0.001 |
| Sex - Male (n, %) | 22201 (45.0) | 27534 (42.4) | <0.001 |
| BMI (mean, SD) | 23.2 (3.5) | 23.2 (3.4) | 0.567 |
| Smoking (n, %) |  |  |  |
| Yes | 7233 (14.7) | 7663 (11.8) | <0.001 |
| No | 42113 (85.3) | 57303 (88.2) |  |
| Alcohol consumption (n, %) |  |  |  |
| <20 g/day | 40413 (81.9) | 52252 (80.4) | 0.300 |
| ≥20 g/day | 8934 (18.1) | 11918 (18.3) |  |
| eGFR, mL/min/1.73m2 (mean, SD) | 72.6 (15.0) | 72.6 (14.2) | 0.937 |
| HbA1c, % (mean, SD) | 5.8 (0.7) | 5.8 (0.6) | 0.421 |
| Fasting blood glucose, mg/dL (mean, SD) | 100.4 (20.9) | 100.0 (18.7) | 0.002 |
| Systolic blood pressure, mmHg (mean, SD) | 130.8 (17.6) | 130.0 (16.4) | <0.001 |
| Diastolic blood pressure, mmHg (mean, SD) | 77.1 (11.1) | 76.9 (10.6) | 0.001 |
| Triglycerides, mg/dL (mean, SD) | 121.8 (83.0) | 119.1 (75.9) | <0.001 |
| HDL cholesterol, mg/dL (mean, SD) | 62.1 (16.7) | 62.9 (16.5) | <0.001 |
| LDL cholesterol, mg/dL (mean, SD) | 123.5 (31.3) | 123.7 (30.4) | 0.297 |
| Urine protein (n, %) |  |  |  |
| Negative | 46735 (94.9) | 62008 (95.4) | <0.001 |
| Positive | 2536 ( 5.1) | 2920 ( 4.5) |  |
| Diabetic medication use (n, %) | 4372 ( 8.9) | 5522 ( 8.5) | 0.032 |
| Antihypertensive medication use (n, %) | 17495 (35.5) | 24487 (37.7) | <0.001 |
| Lipid medication use (n, %) | 10984 (22.3) | 17351 (26.7) | <0.001 |
| Treatment history (n, %) |  |  |  |
| Cerebrovascular disease | 1685 ( 3.4) | 1830 ( 2.8) | <0.001 |
| Cardiovascular disease | 3006 ( 6.1) | 3509 ( 5.4) | <0.001 |

eGFR, estimated glomerular filtration rate; BMI, body mass index
